# Epicardial Fat is Associated with Ventricular Dilation in Patients After the Fontan Operation

**DOI:** 10.1007/s00246-025-04148-1

**Published:** 2026-01-12

**Authors:** Meghan D. Ryan, Nicole St. Clair, Christine K. Lee, Rahul H. Rathod

**Affiliations:** 1 Department of Cardiology, Department of Pediatrics, Boston Children’s Hospital, Harvard Medical School, 300 Longwood Avenue, Boston, MA 02115 USA; 2 Division of Gastroenterology, Hepatology and Nutrition, Department of Pediatrics, Boston Children’s Hospital, Harvard Medical School, Boston, MA USA

**Keywords:** Cardiology, Congenital heart defects, Obesity, Intra-abdominal fat, Magnetic resonance imaging

## Abstract

Epicardial and hepatic fat are suspected to play a role in cardiac remodeling. The primary objective of this study was to identify the cardiac MRI (CMR) parameters that are associated with indexed epicardial fat mass (EFM_*i*_) and hepatic steatosis in patients after the Fontan operation. This was a single-center, retrospective analysis of Fontan patients. Epicardial and subcutaneous fat were analyzed with CMR post-processing software, cvi42 (Circle Cardiovascular Imaging, Calgary, Alberta, Canada). Hepatic steatosis was measured with controlled attenuation parameter (CAP) scores via vibration controlled transient elastography. The cohort included 82 patients (65% male, median age 16 years). On univariate analysis, EFM_*i*_ correlated with BMI (ρ = 0.52, *p* < 0.001), age (ρ = 0.48, *p* < 0.001), ESV_*i*_ (ρ = 0.44, *p* < 0.001), EDV_*i*_ (ρ = 0.42, *p* < 0.001), ventricular mass_*i*_ (ρ = 0.41, *p* < 0.01), subcutaneous fat thickness (ρ = 0.32, *p* < 0.01), ejection fraction (ρ=-0.37, *p* < 0.001), and inferior vena cava (IVC) flow_*i*_ (ρ=-0.26, *p* < 0.05). On multivariable regression analysis, BMI (β 2.21, *p* < 0.001) and EDV_*i*_ (β 0.33, *p* < 0.001) were independently associated with EFM_*i*_ (R^2^ = 0.51). On univariate analysis, CAP scores correlated with BMI (ρ = 0.54, *p* < 0.001), subcutaneous fat thickness (ρ = 0.49, *p* < 0.001), subcutaneous fat mass_*i*_ (ρ = 0.44, *p* < 0.001), age (ρ = 0.36, *p* < 0.01), EFM_*i*_ (ρ = 0.36, *p* < 0.001), and ventricular mass_*i*_ (ρ = 0.24, *p* < 0.05). On linear regression analysis, BMI (β 6.6, *p* < 0.001) was independently associated with CAP scores (R^2^ = 0.27). Neither EFM_*i*_ nor CAP was significantly associated with adverse clinical events. BMI is strongly associated with epicardial and hepatic fat distribution. Epicardial fat mass is also associated with ventricular dilation.

## Introduction

Medical advancements have led to significant improvements in the survival of Fontan patients [1, 2]. However, there are long-term complications due to the chronically elevated systemic venous hypertension and reduced cardiac output inherent in the Fontan circulation. Not only are patients at risk for ventricular dysfunction, but also complications that affect end-organ function [1, 3]. Further, these effects may be compounded by the cardiometabolic consequences of obesity [4, 5].

While the relationship between obesity and cardiovascular disease is well-defined, the influence of visceral and organ-specific adiposity on cardiovascular health is an emerging area of interest. Due to its pro-inflammatory and fibrotic effects, epicardial fat has been implicated in the development of pathogenic conditions, such as coronary artery disease, arrhythmias, and heart failure in the general population [6]. Separately, hepatic steatosis has been found to be an independent predictor of early cardiac remodeling and associated with diastolic dysfunction in adult populations [7, 8]. Techniques for measuring epicardial fat mass are relatively new and primarily involve the use of MRI or CT [6]. Non-invasive techniques for assessing hepatic steatosis have also gained interest in recent years [9]. Controlled attenuation parameter (CAP), a vibration-controlled transient elastography-based technique, provides quantitative prediction of the severity of hepatic steatosis [10].

Fontan patients have significant rates of obesity, with a prevalence comparable to that of the general population [11]. Further, obesity in patients after the Fontan operation has been associated with increased morbidity and mortality [4, 12, 13]. Risk factors for the development of hepatic fat in Fontan patients appear to be similar to those of the general population, such as obesity and dyslipidemia [14]. Although the impact of obesity on Fontan circulation has become an emerging area of interest, there is limited research investigating the influence of epicardial and hepatic fat deposition in this population. Recent work suggests that visceral and epicardial adipose tissue may negatively impact hemodynamics in the Fontan circulation [15, 16]. To further investigate this, we aimed to examine the clinical significance of epicardial and hepatic fat in patients after the Fontan operation.

Our primary objective was to identify which clinical factors and cardiac MRI (CMR) parameters were associated with epicardial fat mass indexed to BSA (EFM_*i*_) and hepatic steatosis. Additionally, we sought to determine the relationships between epicardial, hepatic, and subcutaneous fat. Finally, we studied whether EFM_*i*_ and hepatic steatosis were linked to adverse clinical outcomes.

## Methods

### Patient Selection

This was a single-center, retrospective analysis of patients who have undergone the Fontan operation. Fontan patients were included if they had vibration-controlled transient elastography (VCTE) and CMR within one year of each other. VCTE and CMR studies were performed for clinical surveillance. Exclusion criteria included growth hormone and steroid use due to concern for confounding hepatic fat distribution. Patients were excluded if there were technically inadequate CMR images or CAP measurements needed for analysis. The study was approved by the Institutional Review Board (IRB) at Boston Children’s Hospital.

### Clinical Parameters

Patient demographic and clinical data including cardiac diagnosis, surgical history and laboratory values were abstracted from the medical records. Body mass index (BMI) categories were determined based on CDC growth chart percentiles for patients 2 to 19 years of age and standard adult BMI classifications for patients 20 years and older. BMI classification groups included underweight (< 5th percentile or < 18.5), normal weight (5th to < 85th percentile or 18.5 to 24.9), overweight (85th to < 95th percentile or 25 to 29.9), and obese (≥ 95th percentile or ≥ 30).

Surgical history data included information on all surgeries and catheterization interventions. Major adverse events were also collected, including history of protein-losing enteropathy (PLE), stroke, thrombus, seizures, pleural effusion, and hemoptysis. Arrhythmia history was compiled through a review of Holter monitors, electrophysiology studies, and clinic notes; arrhythmic events included atrial fibrillation, atrial flutter, supraventricular tachycardia, non-sustained ventricular tachycardia (≥ 3 beats lasting < 30 s), sustained ventricular tachycardia (≥ 30 s) and history of cardiac arrest and cardiac rhythm management devices (i.e., pacemakers, defibrillators).

### CMR & VCTE Data Acquisition

CMR studies were performed on a 1.5 Tesla scanner (Philips Healthcare, Best, Netherlands). The details of the CMR protocol have been published previously [17, 18]. Concisely, epicardial fat was measured on an electrocardiogram-gated, steady-state free precession (SSFP) cine image in a short-axis plane from the atrioventricular junction through the apex of the heart. Subcutaneous fat was measured on SSFP axial images at the level of the diaphragm and then confirmed with cross-sectional coronal images. Controlled attenuation parameter (CAP) measurements (dB/m) and liver stiffness measurements (kPa) were measured through vibration-controlled transient elastography (VCTE) using FibroScan^®^ (Echosens, Paris, France). CAP measurements were abstracted from clinical reports. Hepatic fatty infiltration was defined by a CAP score greater than or equal to 215. Consistent with biopsy-validated studies, this CAP score cutoff was used as a high-sensitivity threshold for detecting the presence of steatosis (≥ S1) [19].

### CMR Analysis

CMR data were abstracted from the clinical reports. To account for variations in body size, single ventricle end-diastolic volume (EDV), end-systolic volume (ESV), mass, stroke volume (SV), and inferior vena cava (IVC) flow were indexed to body surface area (BSA) [20–22]. The indexed values are represented by EDV_*i*_, ESV_*i*_, mass_*i*_, SV_*i*_, and IVC flow_*i*_ respectively.

**Fig. 1:**
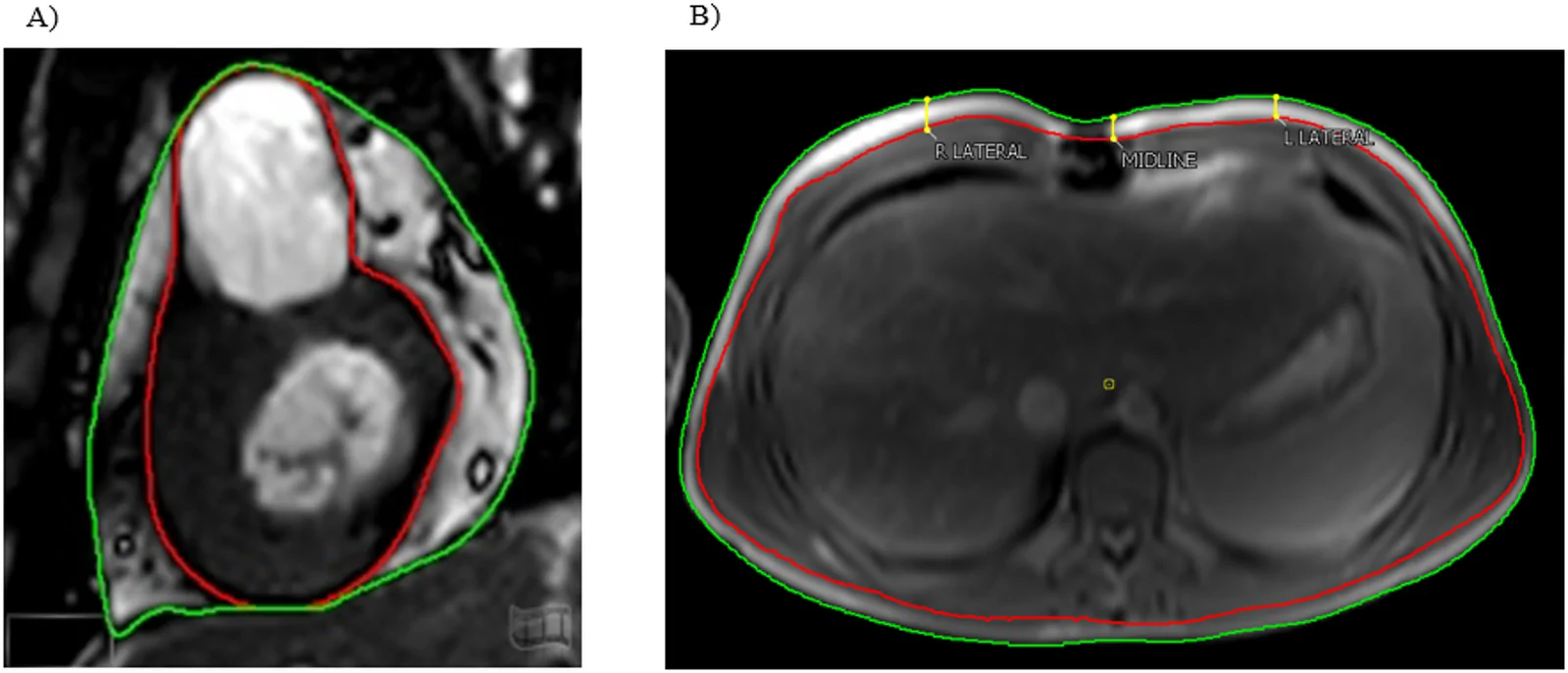
Epicardial fat mass tracing (**A**) and subcutaneous fat mass and linear fat thickness measurements (**B**); post-processing software, cvi42 (Circle Cardiovascular Imaging)

Epicardial and subcutaneous fat were analyzed with CMR post-processing software, cvi42 (Circle Cardiovascular Imaging, Calgary, Alberta, Canada). Two observers independently performed epicardial and subcutaneous fat measurements. Epicardial fat was manually traced with contour tools on consecutive end-systolic short-axis images as shown in Fig. 1a. Cine MRI sequences or dynamic, real-time imaging sequences were used to assess cardiac motion and help delineate epicardial fat from external layers of fat and/or scar tissue. As the software reported ventricular mass using this technique, EFM was calculated using the density conversion between fat (0.9 g/mL) and myocardial tissue (1.05 g/mL) and then indexed to body surface area (EFM_*i*_).

Subcutaneous fat was manually traced on an axial slice at the level of the diaphragm. Similarly, the density conversion between fat and myocardial tissue was used to define subcutaneous fat mass, which was indexed to BSA (SQFM_*i*_), as shown in Fig. 1b. These methods were adapted from Lubert et al., 2018 and Lubert et al., 2020 [15, 16]. Subcutaneous linear thickness was measured perpendicularly at the midpoint between the outer border of the visceral compartment and midline (spinal reference point), as shown in Fig. 1b. The values obtained on either side of the midline were averaged together to find the average subcutaneous linear thickness.

### Statistical Analysis

For data analysis, the Student’s t-test or Wilcoxon rank-sum tests were used to compare continuous variables between two independent groups. Chi-squared test or Fisher’s exact test were used to compare categorical variables. One-way ANOVA tests were used to compare continuous variables between three or more groups. Spearman correlation tests were used to analyze the strength of associations between continuous variables. Multivariable linear regression analyses were used to investigate the associations of EFM_*i*_ and CAP scores with imaging and clinical variables. All p-values were two-tailed with a significance level of *p* < 0.05.

## Results

### Patient Demographics

The study cohort included 82 Fontan patients of which 65% were male (*n* = 53) and 35% were female (*n* = 29). The median age at time of CMR was 16 years [interquartile range: 13 to 19 years]. The median BMI at time of CMR was 21.75 [19.32, 25.62]. The majority of patients were normal weight (*n* = 53, 65%), followed by obese (*n* = 16, 19%), overweight (*n* = 9, 11%), and underweight (*n* = 4, 5%). The patient characteristics of the study population and CMR data stratified by BMI categories are summarized in Table 1.

**Table 1:** Baseline patient and imaging characteristics stratified by BMI groups. (A) demographics and clinical characteristics and (B) imaging and functional parameters _*i*_ = indexed to body surface area

	All patients	Under/normal weight	Overweight/obese	*p*-value
	*N* = 82	*n* = 57	*n* = 25	
	Median [25th, 75th]			
***A***				
Male	53	35	18	0.5
Age	16 [13,19]	15 [12, 18]	18 [16, 23]	**< 0.01**
Weight (kg)	56 [46, 72]	52 [35,58]	77 [72, 90]	**< 0.001**
Height (cm)	160.5 [148, 170]	158 [142, 168]	165 [160, 171]	**< 0.05**
Heart rate (bpm)	83 [72, 94]	80 [72, 94]	85 [74, 90]	0.8
Systolic blood pressure (mmHg)	119 [109,128]	118 [107,127]	123 [110,131]	0.2
Mean blood pressure	84 [77, 92]	82 [75, 91]	88 [83, 95]	**< 0.05**
**Cardiac diagnosis**				0.3
Atrioventricular canal	5 (6%)	5 (9%)	0	
Double inlet left ventricle	7 (9%)	4 (7%)	3 (12%)	
Double outlet right ventricle	11 (13%)	6 (11%)	5 (20%)	
Hypoplastic left heart syndrome	34 (41%)	21 (37%)	13 (52%)	
Mitral atresia (including MA with DORV)	5 (6%)	5 (9%)	0	
Tricuspid atresia	9 (11%)	7 (12%)	2 (8%)	
Other	11 (13%)	9 (16%)	2 (8%)	
**Heterotaxy**	11 (13%)	8 (14%)	3 (12%)	1.0
**Ventricular morphology**				0.1
Left ventricle	21 (26%)	16 (28%)	5 (20%)	
Right ventricle	45 (55%)	27 (47%)	18 (72%)	
Balanced/mixed	16 (20%)	14 (25%)	2 (8%)	
**Surgical history**				
Neonatal surgery	64 (78%)	44 (77%)	20 (80%)	1.0
Stage 2	80 (98%)	56 (98%)	24 (96%)	0.4
Fontan revision	5 (6%)	2 (4%)	3 (12%)	0.2
**Fontan type**				0.06
Lateral tunnel	63 (77%)	40 (70%)	23 (92%)	
Extracardiac conduit	18 (22%)	16 (28%)	2 (8%)	
Hepatic vein to azygous	1 (1%)	1 (2%)	0	
**Comorbidities**				
ECMO	4 (5%)	4 (7%)	0	
Chylous effusions	6 (7%)	4 (7%)	2 (8%)	1.0
Protein-losing enteropathy	3 (4%)	2 (4%)	1 (4%)	1.0
Renal dysfunction	3 (4%)	1 (2%)	2 (8%)	0.2
Seizures	3 (4%)	2 (4%)	1 (4%)	1.0
Stroke	6 (7%)	3 (5%)	3 (12%)	0.4
Thrombus	17 (21%)	12 (21%)	5 (20%)	0.9
Listed for heart transplant	1 (1%)	1 (2%)	0	
Arteriovenous malformations	8 (10%)	5 (9%)	3 (12%)	0.7
Congestive heart failure	6 (7%)	4 (7%)	2 (8%)	1.0
NYHA *≥* 2	13 (16%)	8 (14%)	5 (20%)	0.7
**EP events**				
Arrhythmia atrial	25 (31%)	18 (32%)	7 (28%)	0.8
Ventricular Tachycardia	5 (6%)	3 (5%)	2 (8%)	0.6
Cardiac arrest	6 (7%)	4 (7%)	2 (8%)	1.0
Sinus node dysfunction	5 (6%)	3 (5%)	2 (8%)	0.8
***B***				
**Liver measurements**				
Controlled attenuation parameter (CAP) (dB/m)	205 [176, 239]	185 [163, 210]	265 [227, 301]	**< 0.001**
**CMR measurements**				
Subcutaneous fat mass (SQFM_*i*_)	49 [32, 71]	37 [24, 51]	108 [60, 127]	**< 0.001**
Epicardial fat mass (EFM_*i*_)	61 [47, 75]	54 [44, 70]	71 [57, 92]	**< 0.05**
End-diastolic volume (EDV_*i*_), ml/BSA	108 [93, 129]	108 [94, 129]	108 [87, 129]	1.0
End-systolic volume (ESV_*i*_), ml/BSA	52 [38,67]	52 [38, 66]	51 [40, 72]	0.7
Stroke volume (SV_*i*_), ml/BSA	54 [47, 63]	56 [49, 64]	50 [43, 56]	**< 0.05**
Ejection fraction, %	53 [46, 56]	53 [47, 56]	53 [41, 55]	0.2
Mass_*i*_, g/BSA	55 [44, 66]	54 [43, 64]	59 [52, 73]	0.1
Mass/Volume ratio, g/ml	0.8 [0.5, 1.2]	0.7 [0.5, 1.1]	0.9 [0.5, 1.4]	0.3

*ECMO* extracorporeal membrane oxygenation, *NYHA* New York heart association, functional classification

### Epicardial Fat Mass Analysis

EFM_*i*_ demonstrated strong to moderate, positive correlations with BMI, age, and time since Fontan surgery. There were moderate to weak, positive correlations between EFM_*i*_ and ESV_*i*_, EDV_*i*_, ventricular mass_*i*_, total bilirubin, CAP scores, and subcutaneous fat thickness. There were weak, negative correlations between EFM_i_ and ejection fraction and inferior vena cava (IVC) flow_*i*_. In multivariable linear regression analysis, BMI (β 2.21, *p* < 0.001) and SV EDV_*i*_ (β 0.33, *p* < 0.001) were independently associated with EFM_*i*_ (R^2^ = 0.51) (see Table 3). EFM_*i*_ was not a significant independent predictor of EDV_*i*_. There were no statistically significant relationships between EFM_*i*_ and major clinical events or adverse outcomes. Univariate and multivariate correlation analyses are summarized in Tables 2 and 3.

**Table 2:** Summary of univariate correlation analysis of epicardial fat mass indexed to body surface area

Variable	Correlation coefficient (ρ)	*p*-value (*p*)
Body mass index	0.52	< 0.001
Age	0.48	< 0.001
Time since Fontan surgery	0.45	< 0.001
End-systolic volume_*i*_	0.44	< 0.001
End-diastolic volume_*i*_	0.42	< 0.001
Ventricular mass_*i*_	0.41	< 0.01
Total bilirubin	0.37	< 0.01
CAP scores	0.36	< 0.001
Subcutaneous fat thickness	0.32	< 0.01
Ejection fraction	−0.37	< 0.001
Inferior vena cava flow_*i*_	−0.26	< 0.05

*i* indexed to body surface area, *CAP* controlled attenuation parameter

**Table 3:** Summary of multivariate linear regression analysis of epicardial fat mass indexed to body surface area

Variable	Beta coefficient (β)	*p*-value (*p*)
Body mass index	2.21	< 0.001
End-diastolic volume_*i*_	0.33	< 0.001

*i* indexed to body surface area

### Hepatic Steatosis Analysis

The median CAP score was 205 dB/m [176, 239]. Nearly 40% of patients (*n* = 33) had CAP scores suggesting the presence of hepatic fatty infiltration. Specifically, 24 patients (29%) had CAP scores ranging from 215 to 300 dB/m, representing mild to moderate steatosis, and 9 patients (11%) had CAP scores > 300 dB/m suggestive of severe steatosis. Males had significantly higher median CAP scores than females (225 vs. 196 dB/m; *p* < 0.05). CAP scores demonstrated strong to moderate, positive correlations with BMI, subcutaneous fat thickness, and subcutaneous fat mass_*i*_. There were moderate to weak, positive correlations between CAP scores and age, EFM_*i*_, time since Fontan surgery, and systemic ventricular mass_*i*_. Following linear regression analysis, only BMI (β 6.6, *p* < 0.001) was independently associated with CAP scores (R^2^ = 0.27). No statistically significant associations between CAP scores and adverse clinical events. Univariate correlation and linear regression analyses are summarized in Tables 4 and 5.

**Table 4:** Summary of univariate correlation analysis of controlled attenuation parameter (CAP) scores

Variable	Correlation coefficient (ρ)	*p*-value (*p*)
Body mass index	0.54	< 0.001
Subcutaneous fat thickness	0.49	< 0.001
Subcutaneous fat mass_*i*_	0.44	< 0.001
Age	0.36	< 0.01
Epicardial fat mass_*i*_	0.36	< 0.001
Time since Fontan surgery	0.34	< 0.01
Ventricular mass_*i*_	0.24	< 0.05

*i* indexed to body surface area

**Table 5:** Summary of linear regression analysis of controlled attenuation parameter (CAP)

Variable	Beta coefficient (β)	*p*-value (*p*)
Body mass index	6.6	< 0.001

## Discussion

This study demonstrates that BMI is a strong predictor of both hepatic and epicardial fat distribution. Epicardial fat mass is independently associated with ventricular dilation. Epicardial fat may play an important role in cardiac remodeling and have implications regarding ventricular mechanics.

### BMI-Related Outcomes in Fontan Patients

In our patient population, nearly one third of Fontan patients were overweight or obese, which is consistent with earlier findings on the prevalence of elevated BMIs in other Fontan cohorts [11]. Previous work has established the association between increased BMI and poor outcomes in Fontan patients. Fontan patients with elevated BMI have worse hemodynamics, such as decreased peak oxygen consumption, increased Fontan pressure, and pulmonary capillary wedge pressure [13]. Further, Fontan patients with an elevated BMI are at increased risk for heart failure hospitalization and thromboembolic complications [13]. A recent large, multi-center study (*n* = 3,778, 35 centers) from the Fontan Outcomes Registry using CMR Examinations found that elevated BMI was associated with increased end-diastolic volume and ventricular mass. Fontan patients with obesity had both higher ventricular end-diastolic and Fontan pressures by cardiac catheterization as well [12].

### Role of Visceral Fat Distribution: Epicardial Adipose Tissue

As suggested in the current study, visceral fat distribution may play a role in ventricular adaption. In other patient populations, such as adult patients with heart failure with preserved ejection fraction and pulmonary hypertension, epicardial adipose tissue has been associated with increased risk of morality and worse survival [23, 24].

Our study builds on contemporary work highlighting the growing recognition regarding the clinical significance of epicardial fat in the Fontan population. Lubert et al., 2018 found that Fontan patients have higher epicardial fat volume compared to repaired Tetralogy of Fallot patients. In addition, the researchers observed a weak, inverse correlation between epicardial fat volume and both lower systemic ventricle ejection fraction and cardiac output in Fontan patients [16]. A cross-sectional analysis investigating visceral adiposity and Fontan hemodynamics as measured by cardiac catheterization demonstrated that epicardial fat volume was independently associated with pulmonary vascular resistance [15]. While the two prior studies quantify epicardial fat as volume, the current study intentionally reports epicardial fat as mass. Epicardial fat mass was used as this aligns with standard clinical practice in which solid tissues, such as ventricular myocardium, are reported in terms of mass rather than volume.

The present work contributes to the expanding body of literature, signifying that epicardial fat may play a role in deleterious cardiac modeling as evidenced by changes to end-diastolic volume. Previous research has suggested that changes to ventricular volumes have clinically significant, negative implications on the Fontan circulation. Specifically, a large cohort of Fontan patients demonstrated that increased ventricular dilation as measured by EDV on cardiac MRI was the strongest independent predictor of death or transplant listing in Fontan patients [25]. Further, our study further validates novel methods for measuring epicardial fat using CMR post-processing software [15, 16].

Several mechanisms have been proposed to explain how epicardial adipose tissue may drive alterations in cardiac structure and function. Specifically, epicardial fat contributes to the development of cardiovascular disease through altered gene expression leading to a pro-inflammatory and profibrotic profile as well as interferences in glucose and lipid metabolism [6]. The metabolic activity of local adipose tissue may drive an inflammatory response leading to the release of cytokines that contribute to endothelial dysfunction, myocardial fibrosis, and ventricular remodeling [26].

### Role of Visceral Fat Distribution: Hepatic Steatosis

As alluded to earlier, this study not only focuses on epicardial adipose tissue as a measure of visceral fat, but also hepatic steatosis. Katz et al., 2021 examined the prevalence and associations of hepatic steatosis as measured by abdominal MRI in Fontan patients and found that hepatic steatosis was present in approximately 10% of the study population. Hepatic steatosis was associated with increased BMI and lower HDL cholesterol [14]. As previously demonstrated, obesity remained a strong, independent predictor of the presence of hepatic steatosis in Fontan patients. However, in contrast to the aforementioned study, hepatic steatosis, as defined by a CAP score of 215–300 dB/m, was present in greater than 40% of patients in our cohort. To our knowledge, this is the first analysis of hepatic fat using CAP scores in Fontan patients. This discrepancy in prevalence may be explained by methodological differences between MRI and VCTE as well as the sensitivity thresholds that were applied to define hepatic steatosis.

While hepatic steatosis has previously been linked to early changes in cardiac structure and function, such ventricular remodeling, diastolic dysfunction, and reduced systolic function, the presence of obesity appears to attenuate this relationship [27]. In the current study, ventricular mass_*i*_ was significantly associated with hepatic steatosis on univariate analysis but only BMI remained significant in the final model.

### Limitations

Inherent limitations are introduced by design, as a single-center, retrospective, cross-sectional study. Specifically, the cross-sectional nature of the study prevents the establishment of causal relationships which limits understanding of the mechanisms underlying the identified associations. While referral bias may have led to an overrepresentation of more symptomatic patients undergoing CMR, this is mitigated by our center’s standardized practice of performing CMRs in all Fontan patients once old enough to tolerate imaging protocols. Similarly, in recent years, our center has also started to routinely perform VCTE in patients seen in the Fontan at Boston multidisciplinary clinic. We excluded patients who did not have a CMR and VCTE within 1 year of each other, which may have limited the sample size and led to selection bias, affecting the representativeness of the study population. Further, the limited number of patients who experienced adverse events may have contributed to the absence of significant associations observed, highlighting the need for validation in larger studies. The sample size may have also limited the identification of important independent associations. Specifically, SV EDV_i_ and BMI were independently associated with EFM_i_. However, EFM_i_ was not an independent predictor of SV EDV_i_. Future studies with larger sample sizes may help further elucidate these relationships. Lastly, while CAP scores represent an important, validated non-invasive assessment of hepatic steatosis, interpretation of CAP scores may be confounded by liver congestion and liver biopsy remains the gold standard for determining the severity of fatty infiltration.

### Clinical Significance & Future Directions

This study further contributes to the growing body of evidence outlining the detrimental effects of obesity on the Fontan circulation. Nutritional and exercise-related changes aimed at normalizing BMI may modulate these effects. In our analysis, BMI was the strongest independent association of both epicardial and hepatic fat. By focusing on interventions to address elevated BMIs, visceral fat may also be improved.

Independent of their effects on weight loss and blood glucose control, sodium-glucose cotransporter-2 (SGLT2) inhibitors and receptor agonists have been associated with reduced morbidity and mortality in adult patients with heart failure [28]. Consequently, these medications have garnered interest in the Fontan patient population [29]. Further, glucagon-like peptide-1 (GLP-1) have demonstrated improved cardiovascular outcomes in adult patients with heart failure and obesity [30]. Interestingly, both SGLT2 inhibitors and glucagon-like peptide-1 (GLP-1) receptor agonists have also been shown to cause significant reductions in epicardial adipose tissue in patients with Type 2 Diabetes that may be independent of weight loss [31, 32].

Furthermore, it has been postulated that the pro-fibrotic and inflammatory effects of visceral adipose tissue, such as epicardial fat, may be modifiable and potentially reversible [33]. Epicardial adipose tissue may serve as a promising target for future therapeutic interventions. However, additional research is needed to further establish the clinical significance of visceral fat measures, particularly in the Fontan patient population, and understand the causal mechanisms underlying the associations between epicardial fat mass and ventricular dilation.

## Conclusion

Increased BMI is strongly associated with visceral fat distribution. Epicardial fat was found to be independently associated with ventricular dilation and may be a contributing factor in detrimental cardiac remodeling. Further research is needed to understand the clinical implications of visceral adipose in Fontan patients and potential therapeutic targets.

## Data Availability

No datasets were generated or analysed during the current study.
